# Supplementary material for: The methyltransferase domain of DNMT1 is an essential domain in acute myeloid leukemia independent of DNMT3A mutation
Source: Commun Biol. 2022 Nov 3;5:1174. doi: 10.1038/s42003-022-04139-5 (PMC9633652; doi:10.1038/s42003-022-04139-5)
Supplement: Supplementary file 2 — Supplementary Material [file 42003_2022_4139_MOESM2_ESM.pdf]

Supplementary Information

The methyltransferase domain of DNMT1 is an essential domain in acute myeloid leukemia independent of *DNMT3A* mutation

Balpreet Bhogal, Barbara A. Weir, Ramona Crescenzo, Ann Marien, Min Chul Kwon, Ulrike Philippar, and Glenn S. Cowley

This file includes:

Supplementary Figure 1

Supplementary Figure 2

Supplementary Figure 3

Supplementary Figure 4

Supplementary Figure 5

Supplementary Figure 1

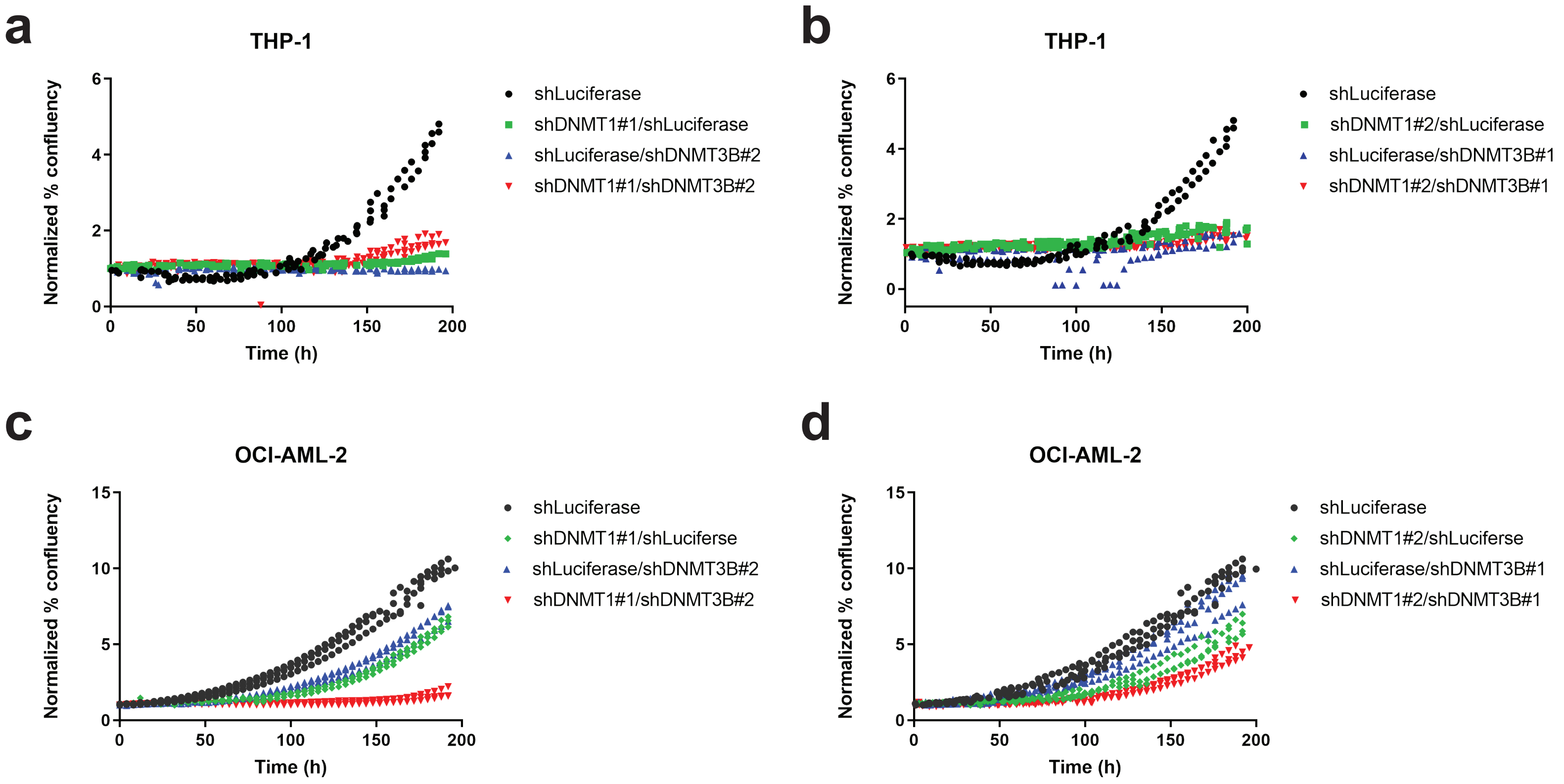

Supplementary Figure 1. (**a-b**) Spheroid-like growth assays on THP-1 cells, a cell line wild-type for *DNMT3A*. (**c-d**) Spheroid-like growth assays on OCI-AML2 cells, which is mutant for *DNMT3A*. Individual values for each replicate are plotted on each graph. Cells were transduced with 2 different combinations of DNMT1 and/or DNMT3B shRNAs. A shRNA targeting the luciferase gene (shLuciferase) was used as a negative control. Spheroid-like confluence size was normalized to the confluence size on day 0 of the assay to calculate normalized % confluency over time. n = 3.

Supplementary Figure 2

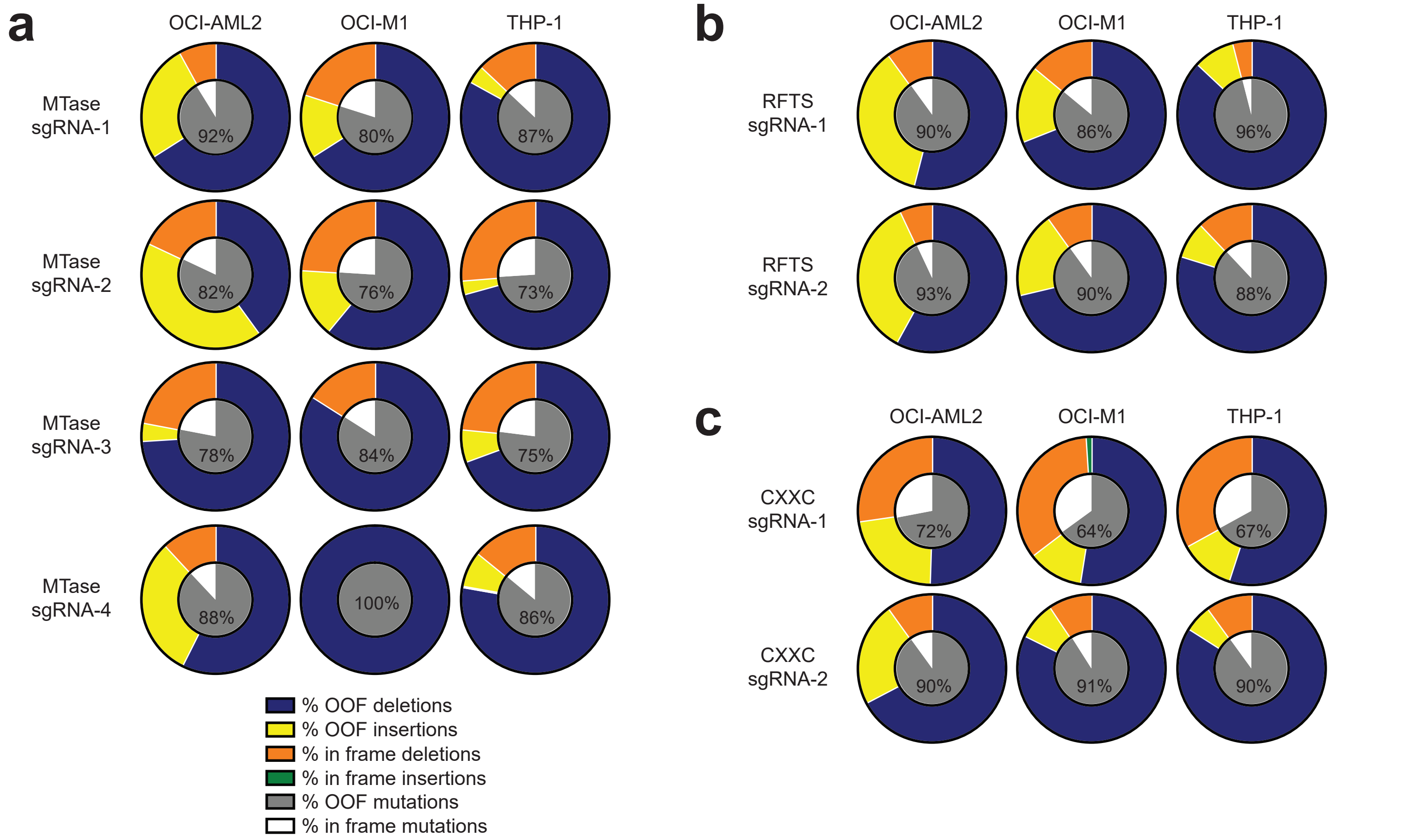

Supplementary Figure 2. Individual sgRNAS targeting *DNMT1* generate comparable mutational spectrums in OCI-AML2, OCI-M1, and THP-1 cells. (**a**) The percentage of out-of-frame deletions (blue), out-o-frame insertions (yellow), in-frame deletions (orange), and in-frame insertions (green) are shown for four sgRNAs targeting the catalytic MTase domain in OCI-AML2, OCI-M1 and THP-1 cells. (**b**) The percentage of out-of-frame and in-frame insertions and deletions are shown for two sgRNAs targeting the RFTS domain in OCI-AML2, OCI-M1 and THP-1 cells. (**c**) The percentage of out-of-frame and in-frame insertions and deletions are shown for two sgRNAs targeting the CXXC domain in OCI-AML2, OCI-M1 and THP-1 cells. The percentage in the center of each circle denotes the total percentage of out-of-frame mutations observed with each sgRNA per cell line.

Supplementary Figure 3

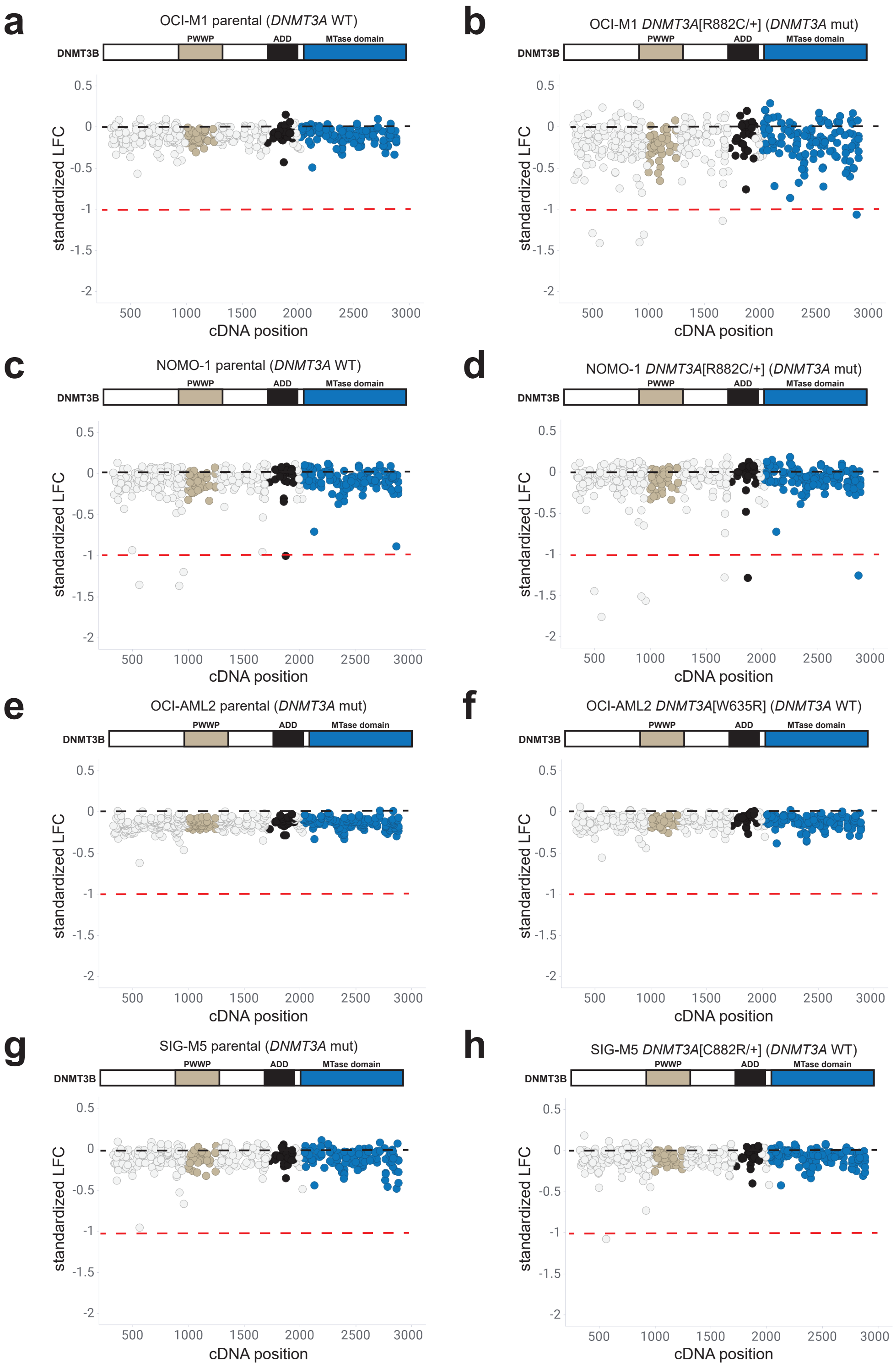

Supplementary Figure 3. Knockout mutations in *DNMT3B* do not significantly impact cell proliferation in OCI-M1, NOMO-1, OCI-AML2, and SIG-M5 isogenic cell lines either wild-type or mutant for *DNMT3A*. **(a-b)** sgRNAs tiling *DNMT3B* are shown with respect to their cDNA position in OCI-M1 parental (*DNMT3A* WT, **a**) and OCI-M1 *DNMT3A*[R882C/+] (*DNMT3A* mutant, **b**) cells. **(c-d)** sgRNAs tiling *DNMT3B* are shown with respect to their cDNA position in NOMO-1 parental (*DNMT3A* WT, **c**) and NOMO-1 *DNMT3A*[R882C/+] (*DNMT3A* mutant, **d**) cells. **(e-f)** sgRNAs tiling *DNMT3B* are shown with respect to their cDNA position in OCI-AML2 parental (*DNMT3A* mutant, **e**) and OCI-AML2[W635R] (*DNMT3A* WT, **f**) cells. **(g-h)** sgRNAs tiling *DNMT3B* are shown with respect to their cDNA position in SIG-M5 parental (*DNMT3A* mutant, **g**) and SIG-M5[C882R/+] (*DNMT3A* WT, **h**) cells. Black dashed bar denotes the mean standardized LFC of the non-targeting controls and the mean standardized LFC of the common essential sgRNAs is denoted by the red dashed line. The color of each circle represents the functional domain it targets: PWWP (light gray); ADD (black); MTase domain (blue). n = 3 replicates for each screen.

Supplementary Figure 4

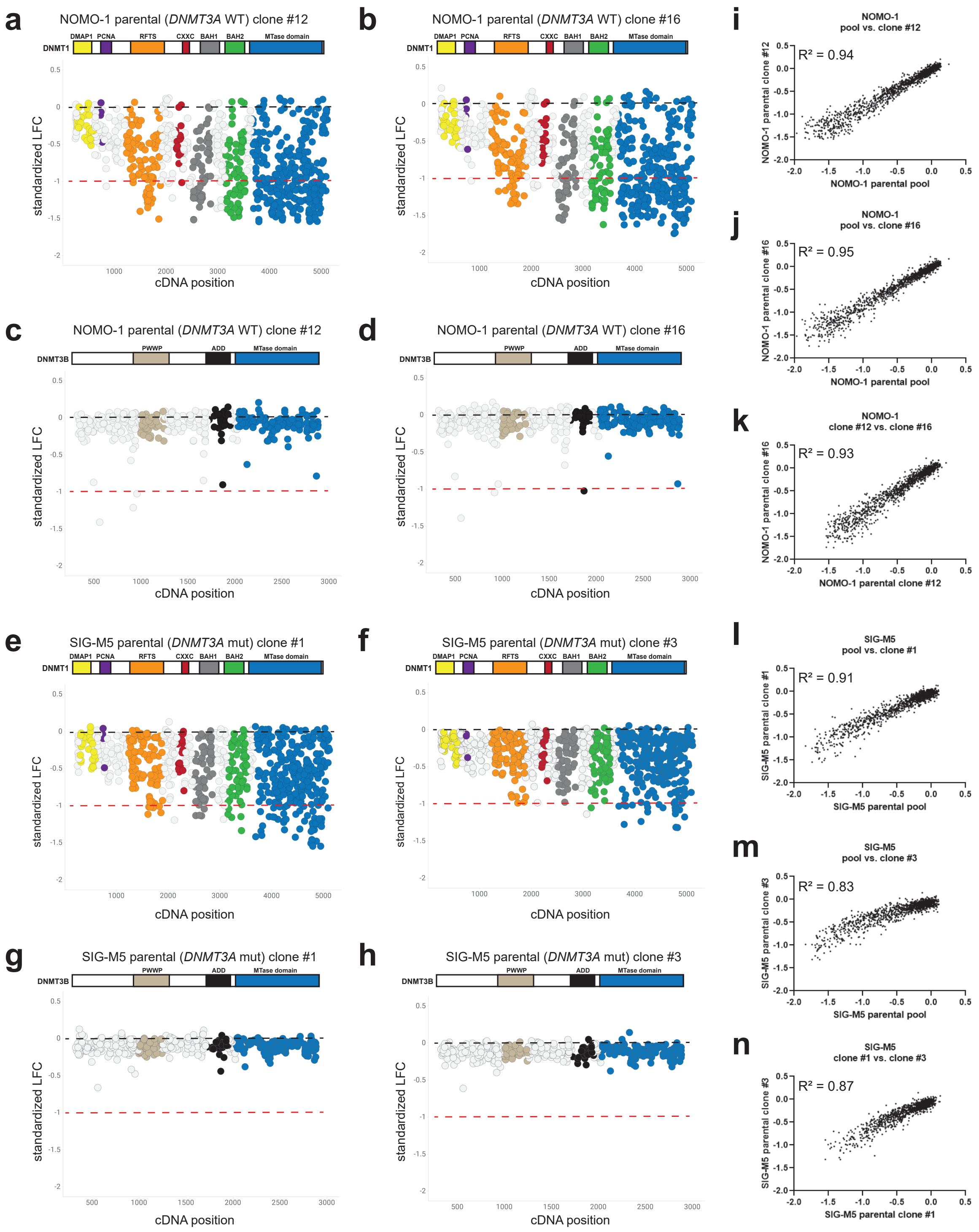

Supplementary Figure 4. Knockout mutations in *DNMT1* and *DNMT3B* have similar effects in NOMO-1 and SIG-M5 parental clones compared to the parental pooled populations. (**a-d**) sgRNAs tiling *DNMT1* (**a-b**) and *DNMT3B* (**c-d**) are shown with respect to their cDNA position along the coding region of each gene in NOMO-1 parental clones. (**e-h**) gRNAs tiling *DNMT1* (**e-f**) and *DNMT3B* (**g-h**) are shown with respect to their cDNA position along the coding region of each gene in SIG-M5 parental clones.Each circle represents an individual sgRNA targeting *DNMT1* or *DNMT3B*. Standardized LFC values for each sgRNA is plotted on each graph. Dashed black bar denotes the mean standardized LFC of the non-targeting controls for each cell line. The mean standardized LFC of the common essential sgRNAs are denoted by the red dashed line. The color of each circle represents the functional domain it targets: DMAP1 (yellow); PCNA (purple); RFTS (orange); CXXC (red); BAH1 (dark gray); BAH2 (green); PWWP (light gray); ADD (black); MTase domain (blue). n = 3 replicates for each cell line screened. (**i-n**) Scatter plots of the standardized LFC values for each *DNMT1* and *DNMT3B* sgRNA across NOMO-1 parental pool and NOMO-1 parental clone #12 (**i**), NOMO-1 parental pool and NOMO-1 parental clone #16 (**j**), NOMO-1 parental clone #12 and NOMO-1 parental clone #16 (**k**), SIG-M5 parental pool and SIG-M5 parental clone #1 (**l**), SIG-M5 parental pool and SIG-M5 parental clone #3 (**m**), SIG-M5 parental clone #1 and SIG-M5 parental clone #3 (**n**). A simple linear regression model was used to calculate the R^2 values for each comparison.

Supplementary Figure 5

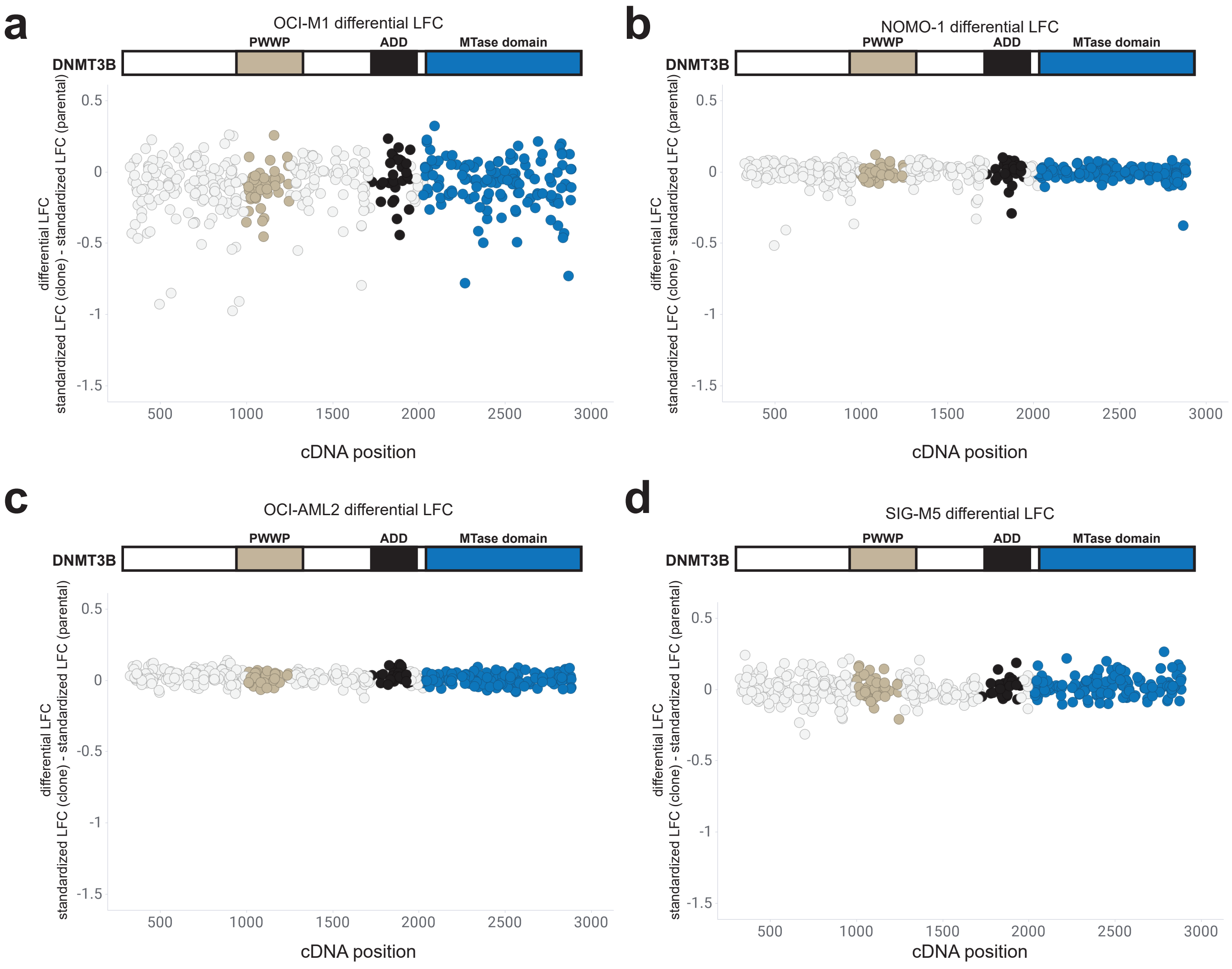

Supplementary Figure 5. AML isogenic cell lines wild-type or mutant for *DNMT3A* are not sensitive to *DNMT3B* knockout mutations. The differential LFC was calculated for each sgRNA across the OCI-M1 (**a**), NOMO-1 (**b**), OCI-AML2 (**c**), and SIG-M5 (**d**) isogenic cell line pairs, using the parental line as a reference. Each circle represents an individual sgRNA spanning the coding region of *DNMT3B*. The color of each circle represents the functional domain it targets: PWWP (light gray); ADD (black); MTase domain (blue). n = 3 replicates for each screen.
