# Supplementary material for: The methyltransferase domain of DNMT1 is an essential domain in acute myeloid leukemia independent of DNMT3A mutation
Source: Commun Biol. 2022 Nov 3;5:1174. doi: 10.1038/s42003-022-04139-5 (PMC9633652; doi:10.1038/s42003-022-04139-5)
Supplement: Supplementary file 3 — Description of Additional Supplementary Files [file 42003_2022_4139_MOESM3_ESM.pdf]

Description of Additional Supplementary Files

File name: Supplementary Data 1

Description: STARS output files for sgRNAs targeting DNMT3B

File name: Supplementary Data 2

Description: Custom DNMT1/3B CRISPR pooled sgRNA library

File name: Supplementary Data 3

Description: Raw counts for NGS results, binning of DNMT1 and DNMT3B CRISPR sgRNAs, and original chip file for custom DNMT1/3B CRISPR pooled sgRNA library.

File name: Supplementary Data 4

Description: Individual sgRNA sequences used for mutational analysis

File name: Supplementary Data 5

Description: Raw data files for data shown in Figure 1.
