# Supplementary material for: The methyltransferase domain of DNMT1 is an essential domain in acute myeloid leukemia independent of DNMT3A mutation
Source: Commun Biol. 2022 Nov 3;5:1174. doi: 10.1038/s42003-022-04139-5 (PMC9633652; doi:10.1038/s42003-022-04139-5)
Supplement: Supplementary file 9 — Reporting Summary [file 42003_2022_4139_MOESM9_ESM.pdf]

nature research

Corresponding author(s): Glenn Cowley

Last updated by author(s): Oct 12, 2022

Reporting Summary

Nature Research wishes to improve the reproducibility of the work that we publish. This form provides structure for consistency and transparency in reporting. For further information on Nature Research policies, see our Editorial Policies and the Editorial Policy Checklist.

Statistics

For all statistical analyses, confirm that the following items are present in the figure legend, table legend, main text, or Methods section.

n/a	Confirmed	
☐	☒	The exact sample size (*n*) for each experimental group/condition, given as a discrete number and unit of measurement
☐	☒	A statement on whether measurements were taken from distinct samples or whether the same sample was measured repeatedly
☐	☒	The statistical test(s) used AND whether they are one- or two-sided<br>*Only common tests should be described solely by name; describe more complex techniques in the Methods section.*
☒	☐	A description of all covariates tested
☒	☐	A description of any assumptions or corrections, such as tests of normality and adjustment for multiple comparisons
☐	☒	A full description of the statistical parameters including central tendency (e.g. means) or other basic estimates (e.g. regression coefficient) AND variation (e.g. standard deviation) or associated estimates of uncertainty (e.g. confidence intervals)
☐	☒	For null hypothesis testing, the test statistic (e.g. *F*, *t*, *r*) with confidence intervals, effect sizes, degrees of freedom and *P* value noted<br>*Give P values as exact values whenever suitable.*
☒	☐	For Bayesian analysis, information on the choice of priors and Markov chain Monte Carlo settings
☒	☐	For hierarchical and complex designs, identification of the appropriate level for tests and full reporting of outcomes
☒	☐	Estimates of effect sizes (e.g. Cohen's *d*, Pearson's *r*), indicating how they were calculated

Our web collection on statistics for biologists contains articles on many of the points above.

Software and code

Policy information about availability of computer code

Data collection	No specialized software or custom code was used for data collection.
Data analysis	Data analysis was performed using the following previously published algorithms and software: STARS (Doench et al. 2016), ProTiler (He et al., 2019) and residual analysis (DeWeirdt et al., 2020).<br>Custom R (version 3.6) code was used for data handling and visualization and is available upon request.

For manuscripts utilizing custom algorithms or software that are central to the research but not yet described in published literature, software must be made available to editors and reviewers. We strongly encourage code deposition in a community repository (e.g. GitHub). See the Nature Research guidelines for submitting code & software for further information.

Data

Policy information about availability of data

All manuscripts must include a data availability statement. This statement should provide the following information, where applicable:

- Accession codes, unique identifiers, or web links for publicly available datasets
- A list of figures that have associated raw data
- A description of any restrictions on data availability

The raw data used to generate plots shown in Figure 1 are available in Supplementary Data 5. The raw count values obtained from the NGS for all DNMT1/3B tiling screens presented in this study are available in Supplementary Data 3. There is a restriction on data availability from the CRISPR genome-wide screens shown in Figure 1. The full dataset is not yet available for publication and we were given approval to show the data for a small subset of genes.

Field-specific reporting

Please select the one below that is the best fit for your research. If you are not sure, read the appropriate sections before making your selection.

☒ Life sciences ☐ Behavioural & social sciences ☐ Ecological, evolutionary & environmental sciences

For a reference copy of the document with all sections, see nature.com/documents/nr-reporting-summary-flat.pdf

Life sciences study design

All studies must disclose on these points even when the disclosure is negative.

Sample size	All screens were performed in triplicate. The good correlation between such screens suggests these are adequate sample sizes.
Data exclusions	For Incucyte studies shown in Figure 1 and Supplementary Figure 1, one replicate from the control and single shRNA samples for the THP-1 studies were excluded from the final analysis due to abnormal growth/spheroid assay visualization that made it difficult to accurately quantify.
Replication	All screens were performed in triplicate with good correlation between such screens.
Randomization	Randomization was not relevant to the current study. All experiments were performed on cell lines and no human/animal cohort was involved.
Blinding	Investigators were not blinded because there was no group allocation for any studies involved here.

Reporting for specific materials, systems and methods

We require information from authors about some types of materials, experimental systems and methods used in many studies. Here, indicate whether each material, system or method listed is relevant to your study. If you are not sure if a list item applies to your research, read the appropriate section before selecting a response.

Materials & experimental systems

n/a	Involved in the study	
☒	☐	Antibodies
☐	☒	Eukaryotic cell lines
☒	☐	Palaeontology and archaeology
☒	☐	Animals and other organisms
☒	☐	Human research participants
☒	☐	Clinical data
☒	☐	Dual use research of concern

Methods

n/a	Involved in the study	
☒	☐	ChIP-seq
☒	☐	Flow cytometry
☒	☐	MRI-based neuroimaging

Eukaryotic cell lines

Policy information about cell lines

Cell line source(s)	ATCC, DSMZ, and the Broad Institute
Authentication	All cell lines used in this study were sent to Genetica for STR and mycoplasma testing several times during the course of our studies.
Mycoplasma contamination	We confirm that all cell lines used in this study were tested for mycoplasma at Genetica/Labcorp and were confirmed to be negative.
Commonly misidentified lines (See ICLAC register)	N/A
